# Variations in achievement of evidence-based, high-impact quality indicators in general practice: An observational study

**DOI:** 10.1371/journal.pone.0177949

**Published:** 2017-07-13

**Authors:** Thomas A. Willis, Robert West, Bruno Rushforth, Tim Stokes, Liz Glidewell, Paul Carder, Simon Faulkner, Robbie Foy, on behalf of the ASPIRE programme team

**Affiliations:** 1 Leeds Institute of Health Sciences, University of Leeds, Leeds, United Kingdom; 2 Foundry Lane Surgery, Leeds, United Kingdom; 3 Department of General Practice & Rural Health, Dunedin School of Medicine, University of Otago, Dunedin, New Zealand; 4 West Yorkshire Research & Development, NHS Bradford Districts CCG, Douglas Mill, Bradford, United Kingdom; 5 NHS Digital, Leeds, United Kingdom; University of the West Indies Faculty of Medical Sciences Mona, JAMAICA

## Abstract

**Background:**

There are widely recognised variations in the delivery and outcomes of healthcare but an incomplete understanding of their causes. There is a growing interest in using routinely collected ‘big data’ in the evaluation of healthcare. We developed a set of evidence-based ‘high impact’ quality indicators (QIs) for primary care and examined variations in achievement of these indicators using routinely collected data in the United Kingdom (UK).

**Methods:**

Cross-sectional analysis of routinely collected, electronic primary care data from a sample of general practices in West Yorkshire, UK (n = 89). The QIs covered aspects of care (including processes and intermediate clinical outcomes) in relation to diabetes, hypertension, atrial fibrillation, myocardial infarction, chronic kidney disease (CKD) and ‘risky’ prescribing combinations. Regression models explored the impact of practice and patient characteristics. Clustering within practice was accounted for by including a random intercept for practice.

**Results:**

Median practice achievement of the QIs ranged from 43.2% (diabetes control) to 72.2% (blood pressure control in CKD). Considerable between-practice variation existed for all indicators: the difference between the highest and lowest performing practices was 26.3 percentage points for risky prescribing and 100 percentage points for anticoagulation in atrial fibrillation. Odds ratios associated with the random effects for practices emphasised this; there was a greater than ten-fold difference in the likelihood of achieving the hypertension indicator between the lowest and highest performing practices. Patient characteristics, in particular age, gender and comorbidity, were consistently but modestly associated with indicator achievement. Statistically significant practice characteristics were identified less frequently in adjusted models.

**Conclusions:**

Despite various policy and improvement initiatives, there are enduring inappropriate variations in the delivery of evidence-based care. Much of this variation is not explained by routinely collected patient or practice variables, and is likely to be attributable to differences in clinical and organisational behaviour.

## Introduction

Clinical and health services research continually produces new evidence that can benefit patients. However, this evidence does not reliably find its way into everyday patient care [1]. This gap between evidence and clinical practice is a strategically important problem for policy-makers, healthcare systems and research funders because it limits the health, social and economic impacts of research [2].

There are particular implementation challenges specific to primary care that are not encountered in other settings. These include rising workloads and demands upon practice: Hobbs et al. have shown substantial increases in both the number and duration of practice consultations in UK primary care over the period 2007–14 [3]. This needs to be considered alongside the complex management of escalating numbers of ageing and multimorbid patients [4], and rising public expectations [5], all in the context of limited practice organisational capacity and continual reforms of general practices [6, 7].

These system strains are compounded by multiple, often competing implementation priorities. In 2012, we identified 107 National Institute for Health and Care Excellence (NICE) clinical guidelines relevant to primary care, as well as 114 specific quality indicators. Together, these yielded a total of 2365 clinical practice recommendations [8]. Several recent studies have identified the problems that may arise when attempting to apply guidelines to patients with multiple morbidities [e.g. 9, 10, 11]. These contextual factors go some way towards explaining the well-documented variations in the delivery of primary care that are consistently observed in the United Kingdom (UK) and internationally [12, 13]. In England, there is evidence that the overall standard of care is improving [14, 15] but the variation between general practices remains [e.g. 16, 17, 18].

‘Big data’ offers considerable promise in healthcare research [19, 20]. One potential opportunity is in the measurement of quality of care delivered across populations. A prime example is the NHS Atlas of Variation in Healthcare [21], which illustrates large geographical variations across England in the level and quality of care in several clinical areas, including diabetes, stroke and cancer. The magnitude of these differences cannot be easily explained by population and casemix factors. Indeed, the Foreword to the Atlas notes that the variation caused by “the idiosyncratic practices of clinicians and of healthcare organisations” represents unwarranted variation (p.15). Variation per se is not necessarily inappropriate; however, the variation examined here is considered ‘inappropriate’ as it means that many patients are generally not receiving recommended care. This is distinct from variation in care where there is not a recommended standard.

Enhanced delivery of care can have substantial implications. For example, it has been estimated that in England more than 7000 strokes per year could be prevented, and 2100 lives saved, with increased adherence to guidelines on the management of atrial fibrillation and use of recommended therapy [22].

Clearly, quality improvement initiatives cannot focus on all clinical practice recommendations at once; there is a need to identify and prioritise those with the potential for the most positive impact for patients. In earlier work [8], we identified a set of evidence-based quality indicators (QIs) based on criteria including: burden of illness (e.g. prevalence, severity); potential for significant patient benefit (e.g. longevity, quality of life); scope for improvement upon current levels of achievement; the extent to which following a recommendation is directly within the control of individual practice teams; and the feasibility of measurement using routinely collected data. This work yielded a set of QIs that we termed ‘high impact’.

We examined achievement against a set of these QIs in a sample of practices in a region of the UK. We aimed to examine the extent to which variations in achievement to high impact indicators can be explained using routinely collected data.

## Methods

### Study design and setting

We conducted a cross-sectional analysis of achievement against selected clinical quality indicators (QIs) using routinely collected, electronic primary care data from a sample of general practices in West Yorkshire, UK. Our analysis included patient and practice characteristics to explore associations with indicator achievement. Data covered the period 1 January 2012 to 31 March 2013, and were extracted during April 2014. The demographic characteristics of the 334 general practices in West Yorkshire are broadly typical of the average across England, with the exception of higher levels of deprivation (practice averaged Index of Multiple Deprivation (IMD) score 29.0 vs 21.8).

### Participants

Of the 334 general practices in West Yorkshire at the time of the study, 272 used the SystmOne clinical information system (TPP, http://www.tpp-uk.com/). This system permits centralised data collection. We sampled randomly from this group and stratified according to the then configured five NHS Primary Care Trusts (PCTs). Assuming a 30% decline rate, we initially approached 78 practices using a conventional, ‘opt in’ approach. After receiving some declines and a small number of acceptances, we decided to approach more practices in order to ensure that our target was reached. We therefore sampled an additional 36 practices, making 114 in total. At the same time, we were granted permission to change to ‘opt out’ recruitment to reduce selection bias by facilitating general practices’ agreement to share anonymised patient data. Ninety three practices did not respond to the initial invitation and were sent a second letter that explained that they could opt out if they did not wish to share data.

### Variables

All clinical data were routinely recorded by practices and amenable to remote extraction. Many of the variables were linked to conditions included in the UK Quality and Outcomes Framework (QOF) year 2012/13. QOF is a pay-for-performance scheme whereby general practices are remunerated according to achievement of targets reflecting the quality of care delivered by the practice [23]. Practice data collection for QOF operates on an annual cyclical basis from 1 April to 31 March. QOF has driven consistent electronic recording of data in general practice and practices routinely record data for incentivised conditions on investigations, prescriptions and intermediate outcomes, such as blood pressure, or glycated haemoglobin [24].

Our dependent variables were drawn from a set of eighteen ‘high impact’ QIs developed by an earlier, multidisciplinary consensus process [8]. The list was reduced to seven indicators (Table 1) following initial data collection and analysis. The level of scope for improvement and potential for intervention were of primary importance in the selection process. Other indicators were rejected due to concerns about the reliability of routinely collected data or uncertainty about the ongoing use of the clinical indicator.

**Table 1:** Details of the clinical indicators included in the study.

Indicator	Description
*Process indicators*	
1. Diabetes: processes of care	A composite indicator recording the proportion of eligible patients that received all of nine recommended processes of care: blood pressure (BP) measurement, glycated haemoglobin (HbA1c) measurement, cholesterol measurement, urine albumin:creatinine ratio (ACR) / protein:creatinine (PCR) testing or proteinuria code, estimated Glomerular Filtration Rate (eGFR) or serum creatinine testing, foot review, retinal screening, body mass index (BMI) recording, smoking status, within the last 15 months (six months for HbA1c measurement).
2. Risky prescribing	A set of nine indicators largely focusing on avoiding adverse gastrointestinal, renal and cardiac effects of non-steroidal anti-inflammatory drugs (NSAIDs) and anti-platelet drugs: prescribing a traditional oral NSAID or low-dose aspirin in patients with a history of peptic ulceration without co-prescription of gastro-protection; prescribing a traditional oral NSAID in patients aged 75 years or over without co-prescription of gastro-protection; prescribing of a traditional oral NSAID and aspirin in patients aged 65 years or over without co-prescription of gastro-protection; prescribing of aspirin and clopidogrel in patients aged 65 years or over without co-prescription of gastro-protection; prescribing of warfarin and a traditional oral NSAID without co-prescription of gastro-protection; prescribing of warfarin and low-dose aspirin or clopidogrel without co-prescription of gastro-protection; prescribing an oral NSAID in patients with heart failure; prescribing an oral NSAID in patients prescribed both a diuretic and an angiotensin-converting-enzyme inhibitor (ACE inhibitor) or angiotensin receptor blocker (ARB); and prescribing an oral NSAID in patients with chronic kidney disease (CKD)
3. Anticoagulation in atrial fibrillation (AF) and risk of stroke	A single indicator examining recommended treatment of patients with AF and at greater risk of stroke. The proportion of patients with a record of AF and a score of two or higher on the CHADS2 risk tool who have a current prescription for anti-coagulation therapy.
4. Secondary prevention of myocardial infarction (MI)	A single indicator showing the proportion of patients with a lifetime record of MI who are receiving the recommended four drugs: ACE inhibitor or ARB, aspirin or antiplatelet, beta-blocker, and statin.
*Outcome indicators*	
5. Diabetes control	A composite indicator recording the proportion of patients with Type 2 diabetes that achieve each of three recommended target levels: BP <140/80 mmHg (or <130/80 mmHg if kidney, eye or cerebrovascular damage), HbA1c ≤59 mmol/mol, and cholesterol ≤5 mmol/l.
6. Blood pressure control in hypertension	A combination of two indicators recording the proportion of patients achieving individual, age-dependent targets in the previous nine months: BP <140/90 mmHg if aged under 80 years, or <150/90 mmHg if aged 80 years or more.
7. Blood pressure control in chronic kidney disease (CKD)	A combination of two indicators assessing the achievement of recommended BP targets in specific patient groups: <140/85 mmHg, or <130/80 mmHg if record of diabetes or proteinuria.

Four indicators focused on processes of care (e.g. prescribing or testing) and three on clinical outcomes (the achievement of recommended targets for the intermediate health outcomes of blood pressure, cholesterol and glycaemic control). Several indicator sets consisted of multiple individual recommendations that were then pooled to permit analysis of achievement. Two of these were composites, assessing the degree to which *all* recommended care processes or treatment targets were achieved for individual patients (e.g. blood pressure, cholesterol and glycaemic values within recommended ranges for patients with Type 2 diabetes [indicator 5: diabetes control]). Others were formed from combinations of individual recommendations, but could not be considered composites in the truest sense as they did not necessarily all apply to a single individual. For example, the risky prescribing indicator (indicator 2) combined nine individual instances of prescribing that might be considered high-risk; six concerned gastro-intestinal risks, two renal risks and one heart failure. These have been detailed elsewhere [25, 26]. Individual patients may have been exposed to one or more of the prescribing risks and the combined measure gave an overall signal of practice risky prescribing. This latter indicator was scored in the opposing direction to the others: lower values here generally suggest safer, more desirable practice, while higher values were desirable on all other indicators. When producing combined indicators, all indicators were equally weighted on the basis that they had all been through a priority-setting process [8] and were considered clinically important.

Independent variables included patient- and practice-level characteristics we hypothesised or recognised from earlier work to be associated with practice performance [27, 28]. At the patient level we examined demographic (age, gender and ethnicity), and illness variables. Illness was assessed by a measure of comorbidity: the number of QOF disease registers on which a patient appeared.

Practice-level variables comprised the number of practice partners (which served as a proxy of practice size), the number of salaried family physicians, and training status—all recorded as at April 2014. We used practice-level Index of Multiple Deprivation (IMD) scores. This measures area deprivation and is determined for each patient on the list, where available, and then averaged over the practice. Overall achievement in the QOF clinical domain (2012–13) was used as a proxy measure for overall quality of care. By providing a wide-ranging and objective measure of items recommended by professionals and patients, it is generally accepted that the QOF offers a good snapshot of quality [29]. Practice prescribing costs over the period were also collected, measured as cost per Age, Sex and Temporary Resident Prescribing Unit (ASTRO-PU) over the year 2012–13.

Two further practice-level variables were included: patient satisfaction (the proportion reporting that they would recommend the practice to others) and practice accessibility (the proportion reporting that they were able to speak with a family physician/nurse within 48 hours of approach).

### Data sources

The NHS Yorkshire and Humber Commissioning Support Unit remotely extracted data from participating practices. The Unit also supplied information on general practice characteristics and practice prescribing data. Patient data were anonymised before transfer to the research team.

Patient satisfaction and practice accessibility data were obtained from the publicly available National General Practice Profiles [30].

### Analysis

We examined sample representativeness by comparing characteristics of participating versus non-participating general practices using the Wilcoxon rank sum test with Monte Carlo permutation sampling. This analysis has been presented elsewhere [31].

Descriptive statistics for the practice and patient variables are presented via means and standard deviations, or median and inter-quartile range where distributions were skewed.

For each clinical indicator we assessed the proportion of cases with documented receipt of appropriate care or target value, as required. Denominators were eligible patients (identified by diagnostic codes and other markers, e.g. prescribing of indicated drugs). Numerators were patients with evidence of a clinical intervention offered or received, or meeting defined treatment targets.

In assessing the impact of practice and patient characteristics, we initially calculated unadjusted odds ratios (ORs) and then adjusted for other variables associated with outcome. We took a parsimonious approach to model selection, retaining significant terms. We added additional variables in a stepwise fashion, with guidance from classification tree and random forest results. We treated ethnicity as a random effects variable to accommodate the large number of categories of ethnicity.

The categories within covariates were formed on a pragmatic basis. Patient age was typically handled as quartiles: <40 years, 40–59 years, 60–79 years, and 80+ years. Other categories were dichotomised using median split (e.g. practice QOF performance, practice accessibility) or approximations of this to provide two meaningful groups (e.g. proportion of salaried family physicians was categorised as 0–25%, 26–100%). Precise categories sometimes differed between indicators because the number of patients varied according to the specific patient group under consideration (e.g. the median value for QOF performance was 645 for diabetes outcomes (~25,000 patients) and 650 for blood pressure control (~78,000 patients)).

It is important to acknowledge that patients are clustered within practices and that practices have an influence over indicator achievement. Logistic regression was used to model achievement with both patient-level and practice-level variables. To account for practice influences beyond the practice-level variables, a random intercept term was added to the logistic regression. The range of odds ratios due to the random intercept was calculated and reported so that the influence of practices expressed by the random intercept can be compared to the associations with other (fixed) variables. Further detail was given by reporting the variance of the practice random intercept as well as the intra-class correlation coefficient (ICC). The ICC can be regarded as providing the proportion of variability due to the general practice.

It is acknowledged that patient assignment to individual practices is likely imperfect and contains measurement error. Practice assignment was taken at the time of extraction and it is therefore assumed that patients were receiving care from that practice throughout the period of interest, but this may not necessarily be so.

Data for almost all patients were complete. Data on age were missing from a small proportion (<1%) of patients and these were excluded from the analysis.

### Study size

Effect size calculations informed a recruitment target of 60 practices. With seven covariates, and a large effect size (defined by a difference of at least 0.8 standard deviations[32]), 60 practices would provide 94% power.

### Ethical approval

The study, including use of opt out recruitment and anonymised, patient-level data, was approved by the NHS Leeds Central Research Ethics Committee (12/YH/0254).

## Results

### Participants

Eighty nine practices (78.1% of those approached) shared patient data. Of the 114 practices originally approached, 22 opted out of data sharing: one had closed, one had merged and it transpired that another provided care for an atypical population. Practices which declined participation only differed from participating practices in having a smaller mean number of family physicians (5 vs. 3.6; *p* = 0.05). The total number of patients in the denominator for each indicator ranged from 4,773 (anticoagulation in atrial fibrillation and risk of stroke) to 77,587 (blood pressure control in hypertension). Patient demography is summarised in Table 2. Practice size was indicated by the number of practice partners (mean [M] 3.7, standard deviation [sd] 2.3) and salaried family physicians (M = 1.3, sd = 1.8). Mean practice-aggregated Index of Multiple Deprivation score was 31.2 (sd = 11.9). Mean QOF 2012/13 performance across practices was 637.4 (sd = 27.6) and 20.2% of the sample were registered as training practices. Mean practice prescribing costs (total Net Ingredient Cost per 1000 ASTRO-PUs [Age, Sex and Temporary Resident Prescribing Unit] were £50.4 (sd = 9.5). Patient reported satisfaction (M = 76.5%, sd = 13.4) and practice accessibility (M = 53.5%, sd = 14.4) were obtained.

**Table 2:** Patient characteristics for cross-sectional analysis of associations with achievement on selected quality indicators.

	Quality indicator						
	1	2	3	4	5	6	7
Number of patients	25 816	37 546	4 773	9 258	25 816	77 587	17 669
Mean age in years (sd)	64.2 (13.6)	71.4 (12.3)	78.96 (8.73)	69.8 (12.1)	64.2 (13.6)	66.7 (13.2)	75.1 (11.39)
Female gender	45.8%	55.8%	49.6%	30.5%	45.8%	52.8%	60.2%
Median number of comorbidities (IQR)	4 (3–5)	3 (2–5)	5 (4–6)	4 (3–5)	4 (3–5)	3 (2–4)	4 (3–5)

sd = standard deviation; IQR = Interquartile range

Quality indicators: 1 Diabetes processes of care, 2 Risky prescribing, 3 Anticoagulation in atrial fibrillation and risk of stroke, 4 Secondary prevention of myocardial infarction, 5 Diabetes control, 6 Blood pressure control in hypertension, 7 Blood pressure control in chronic kidney disease

### Achievement of indicators

**Fig 1:**
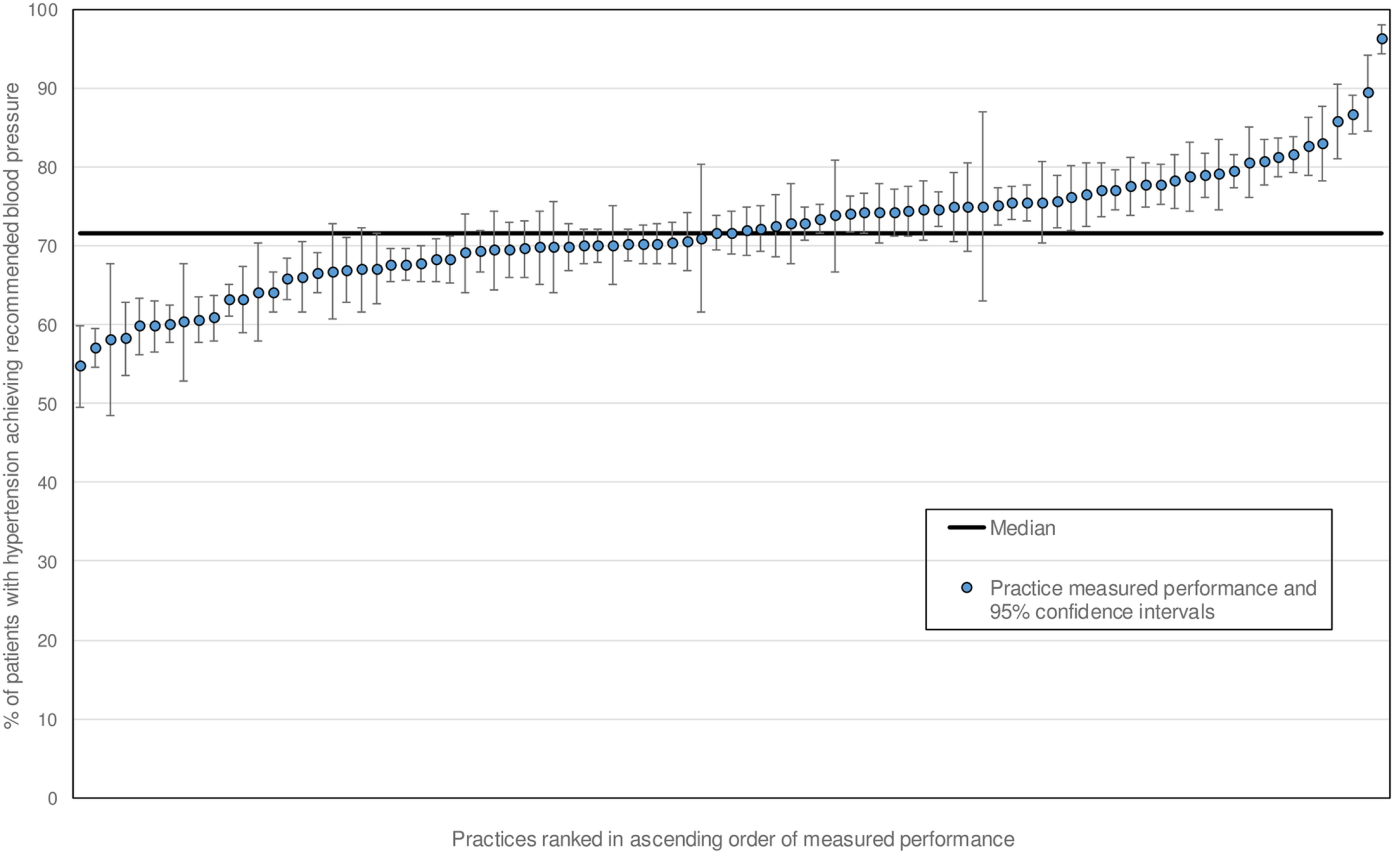
Practice achievement and 95% confidence intervals for blood pressure control in patients with hypertension.

Median practice achievement of the indicators under consideration ranged from 43.2% (diabetes outcomes) to 74.2% (blood pressure control in chronic kidney disease (CKD)) (Tables 3 and 4). Median achievement of the risky prescribing indicator was 8.7%, but this was scored such that a low score was indicative of fewer instances of risky prescribing and was therefore desirable. Considerable between-practice variation in achievement existed on all indicators: the difference between the highest and lowest achievers was 26.3 percentage points for risky prescribing and 100 percentage points for anticoagulation in atrial fibrillation. The variation in achievement of the indicator examining blood pressure in hypertension is presented as an example (Fig 1).

**Table 3:** Practice and patient characteristics significantly associated with process indicators in cross-sectional analysis.

	Diabetes processes of care^1^		Risky prescribing^2^		Anticoagulation in atrial fibrillation and risk of stroke^3^		Secondary prevention of myocardial infarction (MI)^4^	
Number of patients in analysis	25 816		37 546		4 773		9 258	
Median achievement (%)	59.1		8.7		63.0		54.6%	
Range of achievement (%)	8.8–82.3		1.4–27.7		0–100		25.0–83.3	
Range of odds ratios (ORs) for practice residuals	0.09–3.25		0.40–3.51		0.46–1.64		0.70–1.42	
Practice intraclass correlation coefficient (ICC)	0.077		0.056		0.03		0.011	
Practice-level variance	0.276		0.194		0.103		0.036	
Ethnicity variance	0.008		0.004		0.101		0.011	
*Patient variables*	Unadjusted OR (95% CI)	Adjusted OR (95% CI)	Unadjusted OR (95% CI)	Adjusted OR (95% CI)	Unadjusted OR (95% CI)	Adjusted OR (95% CI)	Unadjusted OR (95% CI)	Adjusted OR (95% CI)
Female	1.0	1.0	1.0	1.0	1.0	1.0	1.0	1.0
Male	1.22 (1.16, 1.29)	1.24 (1.17, 1.30)	1.14 (1.06, 1.23)	1.11 (1.02, 1.19)	1.45 (1.28, 1.63)	1.27 (1.12, 1.44)	1.35 (1.24, 1.48)	1.12 (1.02, 1.23)
Age by tertile								
13–59 years	-	-	-	-	1.0	1.0	-	-
60–79 years	-	-	-	-	1.32 (0.93, 1.87)	1.29 (0.90, 1.86)	-	-
80–107 years	-	-	-	-	0.61 (0.43, 0.87)	0.62 (0.43, 0.89)	-	-
Age by quartile								
13–39 years	1.0	1.0	1.0	1.0			1.0	1.0
40–59 years	1.58 (1.39, 1.80)	1.52 (1.33, 1.73)	1.53 (1.00, 2.34)	1.71 (1.12, 2.60)			1.25 (0.73, 2.13)	1.36 (0.79, 2.33)
60–79 years	2.26 (1.99, 2.56)	2.07 (1.81, 2.36)	1.60 (1.06, 2.43)	1.95 (1.29, 2.96)			0.68 (0.40, 1.16)	0.78 (0.45, 1.33)
80–107 years	1.71 (1.49, 1.96)	1.51 (1.30, 1.76)	0.86 (0.56), 1.31)	1.10 (0.72, 1.69)	-	-	0.32 (0.19, 0.54)	0.38 (0.22, 0.65)
Number of comorbidities by tertile								
Lower third	1.0	1.0	1.0	1.0	-	-	1.0	1.0
Middle third	1.34 (1.27, 1.41)	1.24 (1.17, 1.32)	0.81 (0.74, 0.88)	0.81 (0.74, 0.88)	-	-	0.83 (0.76, 0.91)	1.00 (0.91, 1.11)
Upper third	1.49 (1.38, 1.60)	1.33 (1.23, 1.45)	0.55 (0.49, 0.60)	0.56 (0.51, 0.62)	-	-	0.63 (0.56, 0.70)	0.83 (0.73, 0.94)
*Practice variables*								
Proportion of salaried physicians <0.25	-	-	1.0	1.0	-	-	-	-
Proportion of salaried physicians ≥0.25	-	-	0.73 (0.67, 0.78)	0.76 (0.61, 0.94)	-	-	-	-

^1^ Diabetes processes of care. Comorbidity tertiles: 0–3, 4–5, 6–13.

^2^ Risky prescribing. Comorbidity tertiles: 0–2, 3–4, 5–13. **Note**: This indicator scored in the opposite direction, such that lower values generally suggest safer, more desirable practice.

^3^ Anticoagulation in atrial fibrillation and risk of stroke. Data from n = 88 practices as one had no eligible patients.

^4^ Secondary prevention of myocardial infarction. Comorbidity tertiles: 2–3, 4–5, 6–11.

The following practice and patient variables were not significantly associated with achievement: patient-level ethnicity; and practice-level number of practice partners, Index of Multiple Deprivation score (IMD), QOF performance 2012/13, practice training status, patient satisfaction, practice accessibility, and practice prescribing costs.

**Table 4:** Patient and practice characteristics significantly associated with outcome indicators in cross-sectional analysis.

	Diabetes control^1^		Blood pressure control in hypertension^2^		Blood pressure control in chronic kidney disease^3^	
Number of patients in analysis	25 816		77 587		17 669	
Median achievement (%)	43.2		71.7		74.2	
Range of achievement (%)	20.8–66.2		54.7–96.3		50.7–100	
Range of odds ratios (ORs) for practice residuals	0.51–2.05		0.50–5.24		0.54–1.60	
Practice intraclass correlation coefficient (ICC)	0.033		0.039		0.028	
Practice-level variance	0.112		0.134		0.089	
Ethnicity variance	0.0004		0.094		0.043	
*Patient variables*	Unadjusted OR (95% CI)	Adjusted OR (95% CI)	Unadjusted OR (95% CI)	Adjusted OR (95% CI)	Unadjusted OR (95% CI)	Adjusted OR (95% CI)
Female	1.0	1.0	1.0	1.0	-	-
Male	1.03 (0.98, 1.08)	1.09 (1.03, 1.14)	0.80 (0.78, 0.83)	0.86 (0.83, 0.89)	-	-
Age by quartile						
13–39 years	1.0	1.0	1.0	1.0	-	-
40–59 years	1.28 (1.11, 1.48)	1.28 (1.10, 1.47)	1.10 (1.00, 1.22)	1.07 (0.96, 1.18)	-	-
60–79 years	2.59 (2.25, 2.97)	2.55 (2.21, 2.94)	1.37 (1.24, 1.51)	1.22 (1.10, 1.35)	-	-
80–107 years	3.04 (2.62, 3.53)	2.91 (2.48, 3.40)	4.17 (3.74, 4.65)	3.45 (3.08, 3.86)	-	-
Number of comorbidities by tertile						
Lower third	1.0	1.0	1.0	1.0	1.0	1.0
Middle third	1.30 (1.23, 1.37)	1.10 (1.04, 1.17)	1.72 (1.67, 1.79)	1.57 (1.51, 1.63)	0.89 (0.82, 0.96)	0.88 (0.81, 0.95)
Upper third	1.78 (1.66, 1.92)	1.31 (1.21, 1.42)	2.93 (2.79, 3.08)	2.41 (2.29, 2.54)	0.87 (0.80, 0.95)	0.86 (0.79, 0.95)
*Practice variables*						
QOF performance median split						
QOF performance lower	1.0	1.0	1.0	1.0	-	-
QOF performance upper	1.17 (1.11, 1.22)	1.19 (1.02, 1.39)	1.15 (1.11, 1.19)	1.19 (1.02, 1.40)	-	-
Accessibility <54.7%	1.0	1.0	-	-	-	-
Accessibility ≥54.7%	1.12 (1.07, 1.18)	1.18 (1.02, 1.38)	-	-	-	-

^1^ Diabetes control. Comorbidity tertiles: 0–3, 4–5, 6–13; QOF performance median split: <645, ≥645

^2^ Blood pressure control in hypertension. Comorbidity tertiles: 0–2, 3–4, 5–13; QOF performance median split: <650, ≥650

^3^ Blood pressure control in chronic kidney disease. Comorbidity tertiles: 1–3, 4–5, 6–13.

The following practice and patient variables were not significantly associated with achievement: patient-level ethnicity; and practice-level number of practice partners, proportion of salaried family physicians, practice training status, Index of Multiple Deprivation score (IMD), patient satisfaction, and practice prescribing costs.

### Associations with achievement

The range of odds ratios associated with the random effects for practices demonstrate that the likelihood of achieving a specific indicator varied substantially as a consequence of the practice attended (Tables 3 and 4). These ORs were typically of a much greater magnitude than those for other variables, demonstrating strong practice effects. For process indicators, the impact of the practice attended was most pronounced for risky prescribing: a seven-fold difference between the lowest and highest performing practices (OR range 0.40 to 3.51), after adjusting for the variables included in our model. Practice effects were least apparent for secondary prevention of MI (OR range 0.70 to 1.42). There were also sizeable practice effects for outcome indicators. For the achievement of target blood pressure values in hypertension there was a greater than ten-fold difference between the highest and lowest performing practices (OR range 0.50 to 5.24) and for diabetes control the difference was approximately four-fold (OR range 0.51 to 2.05). Practice effects were less marked for the achievement of blood pressure targets in CKD (OR range 0.54 to 1.60). Across the seven indicators, statistically significant associations were identified more frequently with patient than practice characteristics (Tables 3 and 4). The amount of variance explained by these variables, however, was relatively low; practice characteristics explained less than 8% of variance across all seven models. Variance due to patient ethnicity typically explained a small amount of the variance in achievement (less than 10% of the variation due to practice).

#### Process indicators

*Diabetes processes of care*. Males were more likely to receive all nine of the recommended processes of care in diabetes, compared to females (adjusted OR 1.24, 95% confidence interval [CI] 1.17 to 1.30). Relative to younger patients, receipt was more likely in each of the age groups above 40 years old: 40–59 years (1.52, 1.33 to 1.73), 60–79 years (2.07, 1.81 to 2.36) and 80 years and over (1.51, 1.30 to 1.76). Indicator achievement was more likely in those with a greater number of comorbidities: compared with patients appearing on 0–3 QOF registers, the odds were higher for those on 4–5 registers (1.24, 1.17 to 1.32) and 6–13 registers (1.33, 1.23 to 1.45).

*Risky prescribing*. The odds of receiving at least one of the included risky prescribing combinations were greater in males (1.11, 1.02 to 1.19) than females. Risky prescribing was more likely in patients aged 40–59 years (1.71, 1.12 to 2.60) and 60–79 years (1.95, 1.26 to 2.96), but not in those aged 80 years and above, relative to patients under 40 years. Compared with patients with 0–3 comorbidities, risky prescribing was less likely in those on 4–5 (0.81, 0.74 to 0.88) and 6–11 (0.56, 0.51 to 0.62) QOF registers. Attending a practice with a greater proportion of salaried family physicians was also associated with lower occurrence of risky prescribing (0.76, 0.61 to 0.94).

*Anticoagulation in atrial fibrillation and risk of stroke*. Males (1.27, 1.12 to 1.44) were more likely than females to receive the recommended anticoagulation. Patients aged 80 years and older were less likely to do so (0.62, 0.43 to 0.89) than those aged under 60 years.

*Secondary prevention of myocardial infarction*. Males were more likely than females to receive the recommended four medications as secondary prevention of MI (1.12, 1.02 to 1.23). Patients aged 80 years and older were less likely to do so (0.38, 0.22 to 0.65) than those in the youngest quartile. Relative to patients on 0–3 QOF registers, the odds were reduced in patients with higher levels of comorbidity, as indicated by featuring on six or more QOF registers (0.83, 0.73 to 0.94).

#### Outcome indicators

*Diabetes control*. The odds of achieving all three target values in diabetes (BP, cholesterol and HbA1c) were slightly higher in males (1.09, 1.03 to 1.14) than females. The likelihood also increased with age, with all three age groups significantly more likely than patients aged under 40 years to achieve the recommendation (40–59 years: 1.28, 1.10 to 1.47; 60–79 years: 2.55, 2.21 to 2.94; 80 years and above: 2.91, 2.48 to 3.40). A greater level of comorbidity was also associated with indicator achievement: patients on 4–5 QOF registers (1.10, 1.04 to 1.17) and 6–13 QOF registers (1.31, 1.21 to 1.42) were more likely to achieve the targets than those on 0–3 registers. Practices with a better than average QOF performance (1.19, 1.02 to 1.39) and those with better reported accessibility (1.18, 1.02 to 1.38) were more likely to achieve this indicator.

*Blood pressure control in hypertension*. Achievement of target blood pressure values in hypertension was more likely in females than males (0.86, 0.84 to 0.89). The likelihood also increased with age, with patients aged 60–79 years (1.19, 1.07 to 1.31) and particularly those aged 80 years or older (3.34, 2.99 to 3.74) more likely to achieve the indicator than patients aged under 40 years. Compared to patients on 0–3 QOF registers, greater levels of comorbidity were associated with indicator achievement (3–4 QOF registers: 1.54, 1.48 to 1.60; 5–13 registers: 2.32, 2.20 to 2.44). One practice variable was associated with this indicator: achievement was more likely in practices with greater than average QOF performance (1.24, 1.06 to 1.46).

*Blood pressure control in chronic kidney disease*. The only patient or practice characteristic associated with the achievement of target blood pressure in CKD was comorbidity. Target achievement was less likely as the level of comorbidity increased, through 4–5 (OR = 0.88, 95% CI = 0.81, 0.95) and 6–13 QOF registers (OR = 0.86, 95% CI = 0.79, 0.95), respectively.

## Discussion

We found marked variations between general practices in the achievement of clinically important quality indicators. The odds of patients receiving recommended care or achieving recommended treatment targets varied between two- and over ten-fold by indicator according to the practice attended. These marked variations were partly explained by a range of routinely available practice and patient variables; it is likely that much variation is related to clinical and organisational behaviours.

We have demonstrated inappropriate variations for a set of ‘high impact’, evidence-based indicators judged important by clinicians and patients [8]. We did anticipate suboptimal performance given that scope for improvement was an initial selection criterion for the indicators, although we did not know the actual extent of variation. Our findings are generally consistent with previous studies, in England [15, 17, 18] and worldwide [12, 13, 33]. Three of our indicators (diabetes processes and control, anticoagulation in atrial fibrillation) show similar variations in the most recent NHS Atlas of Variation [21]. These levels of inappropriate variation (i.e. unexplained by patient morbidity or preferences) are all the more striking given that several indicators were derived from QOF indicators and NICE guidelines [8]; whilst such initiatives might be necessary for quality improvement they are insufficient by themselves to effectively target inappropriate variations.

One main study strength was the use of opt-out recruitment, whereby sampled practices had to actively decline sharing of their data. This method can produce a larger sample whilst avoiding some of the biases often associated with opt-in recruitment, and thus may be more representative of typical general practices [31, 34].

Our study had several limitations. First, we considered quality of care from a single, technical perspective, i.e. achievement against selected clinical indicators. Other, important aspects of care include interpersonal elements and practice accessibility, and variation has been demonstrated here also (e.g. [13, 35]). Nevertheless, the indicators were the outcome of a rigorous consensus process [8] and we are confident of their importance and relevance to both clinicians and patients.

Second, the sample was restricted to practices using a specific computerised patient record system (SystmOne). Patient record systems are associated with small differences in achievement of incentivised targets [36]. We judged that this by itself was not a major limitation to generalisability as regional coverage was high (over 80% of practices at the time of recruitment).

Third, our study was limited to one geographical area, albeit one with practice characteristics broadly similar to English averages. Moreover, our opt-out approach to practice data sharing has helped ensure that the sample covers a typical range of practices and is likely to have reduced selection bias.

Fourth, routinely collected electronic data offers an efficient but flawed means of gathering large-scale information. Detailed checking of patient records was not possible and it was evident that coding errors were present. In particular, patient ethnicity was poorly recorded: 41% of the patient population in the sampled practices were recorded as ‘British or mixed British’, with a further 14% as ‘unknown’ or ‘ethnic category not stated’. This is a recognised problem with medical records [37] and made analysis of the variable and interpretation of its impact practically meaningless. The quality of research using ‘big data’ is highly dependent upon the quality of data entered, and the extent to which this represents care delivered.

Fifth, the use of combined indicators can mask varying performance between individual component indicators. Giving equal weighting to indicators can also be contentious [38]. However, there is no single agreed method of combining indicators and our methods were similar to those utilised elsewhere [12, 15, 17, 21, 33].

Sixth, we utilised a rather crude measure of deprivation (practice-averaged IMD) that did not consider deprivation at the individual patient level and is likely to have masked differences in variation across practice populations. For reasons of patient confidentiality, we were unable to utilise more precise measures of deprivation.

Given that we were assessing a range of process and outcome indicators concerning different patient populations and clinical behaviours, we would naturally expect different patterns of associations. Nevertheless, we did identify some shared associations, particularly around age, and gender and comorbidity.

Men were generally more likely than women to achieve indicators which is consistent with previous evidence that men tend to receive better care for cardiovascular disease and diabetes [39]. Females, however, were more likely to achieve recommended blood pressure targets in hypertension, and less likely to be the recipient of a risky prescribing combination.

Age had a more varied relationship with our indicators. Older individuals were more likely to receive diabetes processes of care and achieve recommended blood pressure targets. A potential explanation is that older patients with diabetes and hypertension may visit their practice more frequently and thus be exposed to more opportunities for the management of long term conditions. By contrast, two indicators more closely related to prescribing behaviour were less likely to be achieved in older patients. The oldest patients were less likely to receive anticoagulation for stroke prevention in atrial fibrillation. Concerns about frailty and bleeding risk may be relevant here, despite evidence of the benefits of this treatment for elderly patients [40]. Older patients were also less likely to receive recommended treatment for secondary prevention of MI. It may be that prescribing of the four different recommended medications (angiotensin-converting-enzyme inhibitor or angiotensin receptor blocker; aspirin or alternative antiplatelet; beta-blocker; and statin) is less straightforward than in those who are relatively young with fewer comorbidities and contraindications.

Risky prescribing was more likely in patients aged 40–79 years. Short-term prescription of an NSAID without gastro-protection, for example, may be considered less of a risk in younger, otherwise healthy patients. Risky prescribing was also significantly less likely in patients with more comorbidities (i.e. on at least three QOF registers). This result might be considered counter-intuitive if more comorbidities typically means being prescribed more medications, and thus at greater chance of exposure to risky prescription combinations [41]. However, a plausible explanation may be offered for our findings. As suggested in relation to the diabetes results, having more comorbidities—and thus the potential for hazardous medical combinations—may alert health professionals to the risks associated with NSAID prescribing. A study in primary care in Scotland similarly found that risky prescribing was less likely in patients with increasing numbers of repeat medications (as an indication of overall morbidity) [42].

Having a higher number of comorbidities was positively associated with achievement of the diabetes processes, diabetes control, and hypertension target indicators, together with reduced risky prescribing. This finding is consistent with other evidence of a positive relationship between the number of chronic conditions and quality of care [e.g. 43, 44]. The precise nature of the comorbid conditions in question is likely to be an important factor in such a relationship: there is a growing body of evidence to suggest that the presence of diabetes is associated with better preventive treatment of cardiovascular disease [45]. It is perhaps surprising, therefore, to find an inverse relationship between comorbidity and achievement of blood pressure targets in CKD.

Practice-level characteristics showed few associations with indicator achievement and wider evidence for their importance is inconsistent. For example, a systematic review of the impact of practice size upon quality of care suggested that larger practices were associated with improved care, but the evidence was mixed [28]. No association was found between deprivation (measured at the level of primary care organisations) and achievement of diabetes processes or outcomes in the NHS Atlas of Variation [21]. Notably, we found no associations with practice training status although earlier work has suggested an association with features of good quality care [46].

We have conducted parallel, qualitative work exploring health professionals’ views about the determinants of adherence to several of the indicators analysed here [47] shedding some light on the present findings. Interviewees recognised the importance of clinical recommendations from the population perspective, but were also clear that rigid adherence may not be desirable for individual patients (e.g. where someone was already prescribed multiple medications). Attention to the needs of individual patients was considered a general influence across clinical conditions and featured prominently in interviews. The desire to meet these needs (which may be perceived by the clinician rather than actual) was described as a strong driver of prescribing practice, in deciding whether to adhere to recommended targets in diabetes, and in considering whether to prescribe anticoagulation for stroke prevention in atrial fibrillation. Of course, 100% adherence would not be achievable, nor appropriate, but the beliefs of clinical staff may contribute to the observed differences between practices as well as those associated with patient age, gender and comorbidity.

A further possible factor is the role played by organisational culture. Focusing upon the diabetes control indicator, a recent systematic review of qualitative studies [48] offers potential explanations for the recognised between-practice variation. Several of the identified themes concerned professional and organisational components, including resource constraints, deficits in knowledge and skills and uncertainties about professional roles. Thus, higher-performing practices may be those with better structured management systems, access to specialist teams, and shared awareness of guideline recommendations. Elsewhere, the degree of variation observed is suggested to be related “predominantly to the ways in which services for people with diabetes are organised” [21, p.105]. Our finding that practices with higher QOF performance and those with better reported accessibility were more likely to achieve this indicator is consistent with the view that organisation and culture play an important role in performance.

Our work has three messages for the improvement of care and further research. First, the consistent, substantial variation between practices emphasises that this remains an important issue, with variation existing at similar levels to that assessed almost two decades ago [13]. It is concerning that the likelihood of receiving recommended care at different practices can vary by such magnitude. There remains a need to better understand the reasons behind these differences and develop interventions to narrow the gap between the lowest and highest performers. A small number of studies have examined variation at multiple levels, i.e. that occurring between practices and that between practitioners within practices [42]. This has shown how the role played by different types of variation differs by the outcomes under observation. Unfortunately, this level of analysis was not possible in the current study as individual clinicians could not be identified.

Second, the modest but significant associations between achievement and specific patient characteristics have implications for improvement strategies. Better performance on the diabetes, hypertension and risky prescribing indicators was associated with comorbidity. Thus, greater attention could be focused upon patients who are (relatively) healthier and consequently less likely to attend the practice. We notably did find an association between practice accessibility and achievement of the diabetes control indicator.

Third, the associations between patient and practice variables and indicator achievement suggests the importance of clinical and organisational behaviours. Qualitative research has shown that health professionals often believe that practice performance is heavily influenced by local casemix or demographic variables [47]. Research such as our own can combat such potentially erroneous perceptions. The potential importance of organisational culture is recognised [35, 49]. Our recent qualitative work also recognised the importance of internal practice norms and ways of working, e.g. shared understanding of professional roles and the delegation of responsibilities for different tasks and management decisions [47]. Partly due to difficulty of measurement, organisational culture or climate is often not considered but warrants further investigation. Further work is needed to elucidate the reasons for practice-level variation and to design and evaluate interventions targeting practice teams to encourage consistent delivery of evidence-based clinical care.

Several interventions to promote guideline implementation (e.g. audit and feedback, educational outreach) have modest if variable effects [50, 51]. We are presently conducting a cluster randomised controlled trial of an intervention to improve achievement of a subset of high impact indicators by targeting identified barriers to change [52].
